# The zone model: A conceptual model for understanding the microenvironment of chronic wound infection

**DOI:** 10.1111/wrr.12841

**Published:** 2020-06-30

**Authors:** Klaus Kirketerp‐Møller, Philip S. Stewart, Thomas Bjarnsholt

**Affiliations:** ^1^ Copenhagen Wound Healing Center Bispebjerg University Hospital Copenhagen Denmark; ^2^ Center for Biofilm Engineering Montana State University Bozeman Montana USA; ^3^ Costerton Biofilm Center, University of Copenhagen and Department of Clinical Microbiology Copenhagen University Hospital Copenhagen Denmark

## Abstract

In 2008, two articles in Wound Repair and Regeneration changed the clinical perspective on chronic wounds. They stated that chronic wounds that do not heal contain bacterial biofilms and that these biofilms may be one of the reasons for the nonhealing properties of the wounds. However, we still do not understand the exact role biofilms play in the halted healing process, and we are not able to successfully treat them. The reason for this could be that in vivo biofilms differ substantially from in vitro biofilms, and that most of the knowledge about biofilms originates from in vitro research. In this article, we introduce the zone model as a concept for understanding bacterial behavior and the impact of the microenvironment on both the host and the bacteria. Until now, identification of bacteria, gene expression, and postscript regulation have been looking at a bulk of bacteria and averaging the behavior of all the bacteria. As the zone model dictates that every single bacterium reacts to its own microenvironment, the model may facilitate the planning of future research with improved clinical relevance. The zone model integrates physiology and biology from single cells, microbial aggregates, local host response, surrounding tissue, and the systemic context of the whole host. Understanding the mechanisms behind the actions and reactions by a single bacterium when interacting with other neighboring bacteria cells, other microorganisms, and the host will help us overcome the detrimental effects of bacteria in chronic wounds. Furthermore, we propose use of the terminology “bacterial phenotype” when describing the actions and reactions of bacteria, and the term “biofilms” to describe the morphology of the bacterial community.

## Graphical abstract

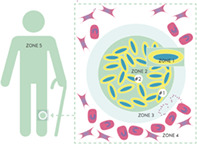

## 1 INTRODUCTION

In 2008, James et al^1^ and Bjarnsholt et al^2^ provided the first direct evidence that bacterial biofilms are present in chronic wounds and suggested that these might contribute to the nonhealing state of these wounds. These articles have been cited more than 600 times (unique citations, PubMed August 2019), but a change in clinical practice and outcome is absent. Why?

There is not a single straightforward answer to this question. One possibility is that the cause and effect relationship between biofilm and chronicity is difficult to prove. Another is that practical diagnostics for the presence of biofilm in a wound are lacking. A third possible answer could be a lack of complete knowledge and/or misconceptions. Misinterpretation of current knowledge is driven by the assumptions and interpretation that newly‐gained scientific information creates. This article is an opinion article, highlighting some of the caveats and misinterpretations in current research, and proposing the zone model as a path toward a holistic perspective on the role of bacterial burden/infections in impairing healing of chronic wounds.

As a concrete example of an existing misconception, consider that, when we (scientists and a clinician) started our research on bacterial biofilm in chronic wounds in 2005, we were often met with the belief that biofilm is the slimy surface of wounds and that it can easily be removed by debridement. Present beliefs appear no different; many (if not most) clinicians, including both medical doctors and nurses, interpret the visible surface as the biofilm. It has been shown, however, that the surface is not a confluent layer of bacteria, but rather that microorganisms can be found deeper in wound tissue.^3^, ^4^ Superficial debridement does not remove all of the biofilm.

James et al^1^ observed that 60% of chronic wounds examined contained biofilms, which were detected by confocal and electron microscopy. Furthermore, it was found that the biofilm infections were composed of many different bacterial species, supported by standard culturing and metagenomics. Evidence for multispecies infections was likewise supported by the work of Bjarnsholt et al^2^ (although multispecies biofilms were not). The presence of multispecies biofilms is still debated, but a review by Burmølle et al^5^ demonstrates a trend in biofilm infection toward lower bacterial diversity of bacteria and strictly mono‐species biofilm. This shift toward a lower diversity in pathological biofilms is confirmed by a recent study of the skin microbiota by Ring et al.^6^

The discussion on multi or mono‐species biofilms may also be blurred due to a lack of definition of what constitutes a biofilm. Two different concepts may be valid:

Concept A—Each microbial aggregate contains a single species. There may be distinct spatially separated aggregates of many different species. But, if each aggregate is considered to be a separate biofilm, it is clear that the biofilms are not multispecies. There is a constellation of separate biofilms that likely interact with each other chemically if they are close enough.

Concept B—Each microbial aggregate contains a single species and, as above, there may be distinct aggregates of many different species. If one conceives of the biofilm as this collective group of aggregates, along with the host material and tissue in which aggregates are distributed. There is one biofilm and it is mixed‐species.

The article by Bjarnsholt et al^2^ found similarities between the etiology of the chronic lung infection of cystic fibrosis patients and chronic wounds with respect to biofilm formation and accumulation of polymorphonuclear leukocytes (PMN) around the bacterial biofilms. There were also indications that the PMNs exhibited impaired antimicrobial function in the vicinity of the biofilms. It was proposed that this lack of efficacy was due to PMN killing by secreted virulence factors, such as rhamnolipid produced by *Pseudomonas aeruginosa*.^7^ The lysis of a PMN by rhamnolipid results in the entire arsenal of the lysed PMN being released into the tissue. This probably causes collateral damage and thereby the attraction of more PMNs. This could explain, at least partly, the inflammatory chronicity of the wounds, as well as the composition of the wound fluid and accumulation of PMNs in the wound tissue. The latter has previously been described by Falanga^8^ as the chronic wounds are trapped in an inflammatory state. The chronicity of the infection seems to launch both the innate and the acquired immune defense responses in the host. These are incapable of eliminating the bacteria and cause collateral damage.^9^

The most important discovery from the two first articles (to visualize biofilms in chronic infections) is that bacterial biofilms do exist in chronic wounds, despite the absence of an abiotic surface to which the bacteria can attach. The definition of a bacterial biofilm at the time these articles were published was still “aggregates of bacteria, embedded in a extracellular polymeric substance, irreversibly attached to a surface.”^10^, ^11^ The notion of biofilms without a surface attachment was supported by a meta‐analysis by Malone et al,^12^ which found that 78% of all chronic wounds contained bacterial biofilms. Due to the fact that biofilm aggregates in chronic wounds are heterogeneously distributed^5^ and not all samples contain bacteria, the actual incidence of biofilm presence in chronic wounds may be higher.

Biofilm infections are difficult to eradicate and many treatment possibilities in chronic wounds have been suggested. These include antibiofilm strategies^13^ and local antimicrobials like silver.^14^ Most of these therapies are based on in vitro testing. Very few have been proven or tested in humans, and even fewer in randomized controlled trials. A key challenge in clinical practice is that of eradicating bacterial biofilms in chronic wounds and all other chronic infections. We propose that the lack of understanding of biofilms and of their microenvironment in chronic infections prevents us from locating the correct research target because we cannot see the forest for the trees. In this article, we propose the zone model in order to better understand the behavior of bacteria in biofilms interacting with the host in the context of a localized infection.

## 2 WHAT DO WE KNOW ABOUT BACTERIA IN CHRONIC WOUNDS?

The sampling method itself affects the identification of bacteria in chronic wounds. A standard swab will pick up superficial bacteria while a biopsy will be able to sample bacteria located deeper in the tissue. Culturing of bacteria in selective or nonselective growth media is still a clinical reality in most of the world. Culturing depends on the ability of the specific bacteria to grow on selected media and under different environments (ie, aerobic or anaerobic). Fastidious and slow‐growing bacteria may not be detected due to lack of the correct nutrients in the agar or too short observation time. In addition, viable but nonculturable bacteria, as well as dormant bacteria (as seen in biofilms) are often not detected by routine culturing.^15^, ^16^ Molecular methods have emerged, including next‐generation sequencing for metagenomics.^15^, ^17^ Despite the fact that we are now able to identify more organisms in chronic wounds, the recognition of relevant pathogens still relies on a professional guess.

RNA sequencing identifies the microorganism and is also able to elucidate the activity.^18^ This method is predominantly used in research and will (eventually) reveal bacterial behavior, strategies, and processes during infections. This will give us a better understanding of the nature of infections, including chronic/biofilm infections. Whatever the method, identification of microorganisms from clinical samples has one major limitation. The results will only reveal microorganisms present in the specific sample analyzed and not in the entire area of interest. RNAseq can give us information regarding ratios of activity in the entire community of microorganisms, but not information on individual bacteria All the techniques mentioned here require a sample from the site to be examined and, as explained below, the sample site will have an impact on which pathogens are found.

## 3 WHAT DO WE KNOW ABOUT BIOFILMS IN CHRONIC WOUNDS?

Biopsies taken from different parts of wounds contain highly variable numbers and types of bacteria.^15^, ^19^ Bacteria in chronic wounds are heterogeneously distributed (eg, *Staphylococcus aureus* prefer to settle nearer to the surface than *P aeruginosa* ^3^, ^4^). This most likely occurs due to local environmental differences in nutrition, oxygen concentration, and host response. Gradients of these factors are also seen within bacterial biofilms.^20^ These findings suggest that different bacteria prefer different environments and/or that they are in competition with other bacteria and find niches in chronic wounds where they have the best survival opportunities.

Although multispecies aggregation is described in the original article by James et al^1^ and has been cited in many articles, few subsequent studies support this finding.^21^ The majority of clinical biofilm studies report mainly mono‐species aggregates of biofilm.^5^, ^22^, ^23^ Bjarnsholt et al^24^ conducted a systematic review of the size of the biofilm aggregates in clinical biofilm infections. The dimension of the biofilm in chronic wounds ranged from 5 to 200 μm and, as such, were far from the size of the biofilm models in a flow‐cell or micro‐titer dish that may be similar in thickness, but the lateral dimension is very different. The origin of individual bacterial biofilm could also determine single‐ or multi‐species biofilm formation. If a single planktonic bacterium manages to settle in unoccupied tissue and multiply, the result is most likely a single‐species biofilm. Previous findings suggest that it is likely that clumps of bacterial biofilm are introduced into the tissue, settle, and adapt.^25^ The likelihood of this being a single‐species biofilm is high due to the competitive nature of bacteria. If there is, however, an abundant local supply of nutrients, such as in areas of wounds with necrotic tissue, bacteria could evolve side‐by‐side or even within the same biofilm.

Roberts et al^26^ concluded that despite the fact that in vitro models have provided useful information to aid the understanding of biofilms, they do not represent the in vivo situation. As the in vitro biofilm often covers the surface of the flow‐cell or the micro‐titer plate, it again contributes to the mistaken idea that the slimy surface of a chronic wound is a biofilm, and that it can be readily removed or targeted.

### 3.1 Bacterial behavior

Cornforth et al^18^ demonstrated that *P aeruginosa* behaves differently when grown in vitro compared to bacteria taken directly from animal or human specimens. They showed that whether the environment is human, another animal, or in vitro can be discerned based on transcriptomic patterns of gene expression. Whether in vitro models (or even animal models) capture the human clinical reality closely enough to be translationally useful is thus questionable. We need to address this issue and acknowledge that bacteria alter their behavior according to challenges and opportunities in their environment. Since the bacteria are embedded in matrix, every single bacterium has its own unique environment, influenced by the local availability of oxygen, specific nutrients molecules, and myriad host factors and cells.

### 3.2 Wound fluid analysis

Analysis of the wound fluid has the potential to give us information about the microorganisms present, their excreted products, and their effect on the host response at a humoral, proteolytic, and cellular level. However, numerous factors influence the results and the interpretation of these assays.^27^ These factors include sampling techniques, sample processing, and analysis methods. Wound fluid often contains high amounts of proteolytic enzymes, many of which are activated, and this will alter the composition of the wound fluid prior, during, and after the sampling. It has been demonstrated in several articles that proteolytic enzymes like the matrix metalloproteases are elevated in chronic wounds.^28^, ^29^, ^30^, ^31^

The interpretation of the wound fluid is limited by the fact that the composition of the fluid is an average of the sample site. Hence, it will not be able to give us information from specific sub‐areas of interest in the wound. Wound fluid can be collected at a specific time point, for example, along with the swab used to collect bacteria, but usually the collection of wound fluid is carried out over hours.^32^, ^33^ In this way, the wound fluid is both an average of the fluid from the entire wound and an average over time.

## 4 INTRODUCING THE ZONE MODEL

**FIGURE 1:**
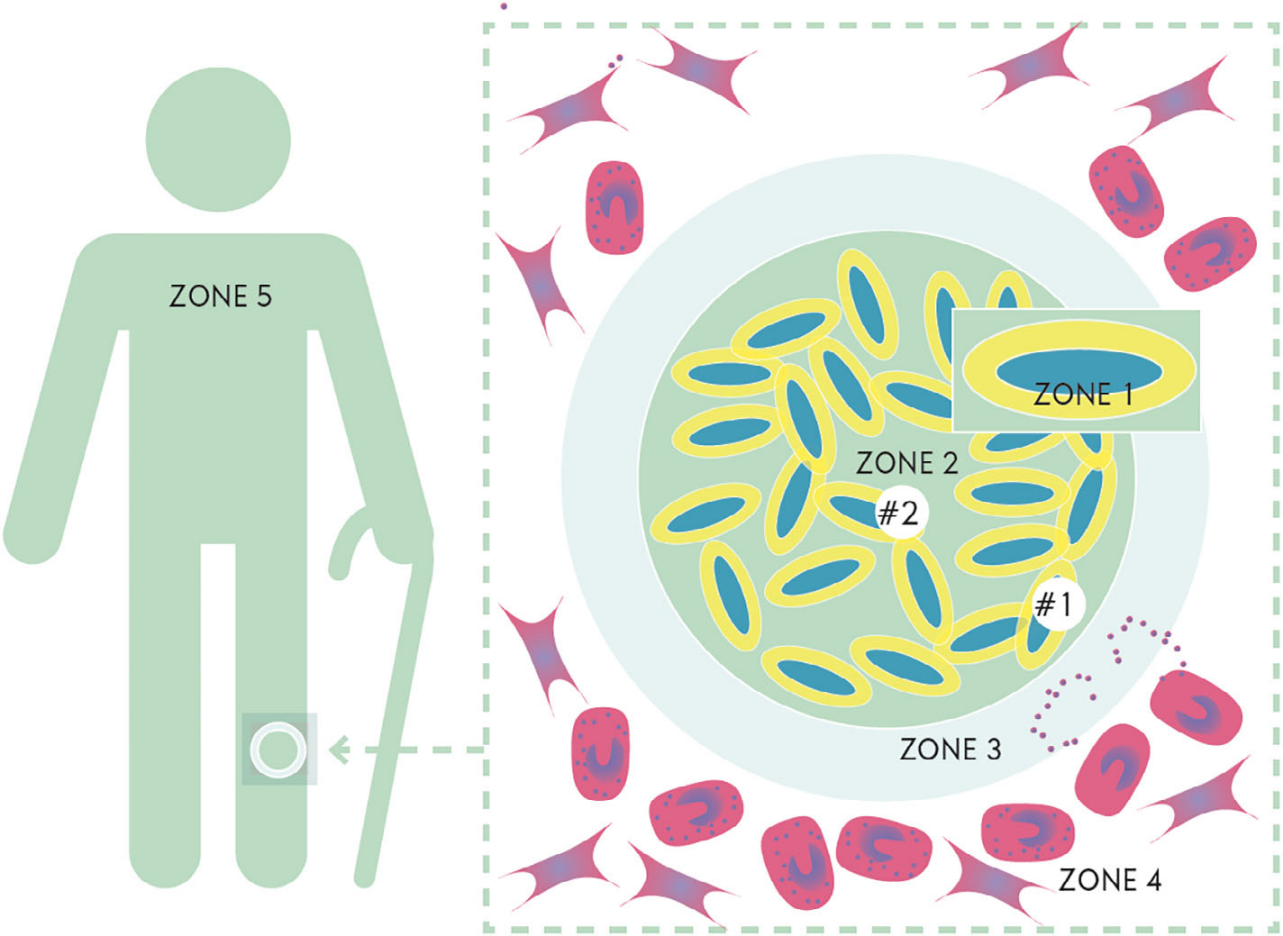
The Zone Model. Zone 1: The exocapsular zone. This is the zone just adjacent to the bacteria. It influences the behavior of the bacteria. Excretion of bacterial products are delivered here. Zone 2: The Biofilm. This is the bacteria together with the extracellular matrix. Zone 3: The Exobiofilm zone. This is the zone where interaction with the host is most pronounced (the ‘combat zone’). Zone 4: The environmental zone. This is the wound bed where host cells try to establish healing of the wound. Cells like fibroblasts, neutrophils, macrophages, lymphocytes, and keratocytes clear the tissue of debris, dead host cells, and bacteria; these cells deposit collagen as a scaffold for wound repair. Zone 5: The host. Immune response cells are produced far from the wound and are delivered to the wound area by the blood stream. Numerous factors in the host will affect delivery and efficacy of this response. Diabetes, arteriosclerosis, or acquired defects in the immune system are just a few examples of factors that may affect this process

To improve how we understand and research biofilms in chronic wounds, we introduce the zone model. It is important to realize that many chronic wounds heal with proper traditional treatment. Venous leg ulcers need compression therapy, and pressure ulcers and diabetic foot ulcers needs off‐loading. Many chronic wounds are chronic mainly due to insufficient treatment, despite containing bacteria in biofilms. Despite this, most chronic wounds will heal with correct conventional therapy. As many chronic wounds with biofilms heal, we have to consider if the biofilms, per see, disturb the healing. Most likely, the extracellular matrix does not affect healing. The phenotype of the bacteria might obstruct healing by producing virulence factors^9^ and thereby changing the microenvironment surround the bacteria. The microenvironment is important for the phenotype of the bacteria. In this context, we refer to the immediate microenvironment that surrounds the bacteria, as can be seen in the zone model (Figure 1).

Zone 1 is specific to the single bacterium. This environment determines the physiological state of the bacterium based on oxygen level, carbon sources, antimicrobial factors, and so on. The entire genome (and transcriptome) of the single bacterium, together with the microenvironment in zone 1, determines the phenotype of the bacterium. zone 1 is small and difficult to study. If bacteria are situated in the center of the biofilm, it is likely that oxygen, iron, and other nutrients are depleted. Below a certain threshold, the bacterium becomes dormant, awaiting a change in its zone 1 environment.

Zone 2 is the bacterial aggregate itself. This is composed of multiple bacteria embedded in a matrix (which we assume is produced by the bacteria itself) and intermixed with polymers (eDNA, proteins, and polysaccharides) from the host. Oxygen concentration declines toward the center of the biofilm, and it is assumed that this could also be true for certain antimicrobials, nutritional factors, and so on.

Zone 3 is the environment encapsulating the aggregate. Here the host reaction against the bacteria is delivered. The bacterial defense against the host is also deployed here. It could be referred to as the “Combat Zone.” The effect of the collateral damage^9^ is most profound in this zone. It is from this zone that oxygen, nutrition, antimicrobial agents, and so on are delivered to the surface of the aggregate.

Zone 4 is the surrounding wound tissue. This zone reflects the predisposing (host) factors and the systemic impact of the fight between bacteria and host. Zone 4 is not the same for all bacterial biofilms in chronic wounds. In the superficial layer of the chronic wound the density of bacteria is high, but in the deeper parts of the wound the distance between biofilm aggregates is larger. It has been postulated that biofilm may be left behind in tissue even after removal of all granulation tissue.^34^ The composition of zone 4 may be responsible for the uneven distribution of bacteria and species within the wound tissue. In zone 4, the host immune cells, such as PMNs, macrophages, and (T‐ and B‐) lymphocytes, are embedded in a scaffold of collagen and fibroblasts of the granulation tissue. If we accept concept A (mentioned in the introduction), zone 4 is harboring distinctly different biofilms and is separating these. Collaboration, mutualism, and commensalism between the different biofilms may happen in zone 4.

**FIGURE 2:**
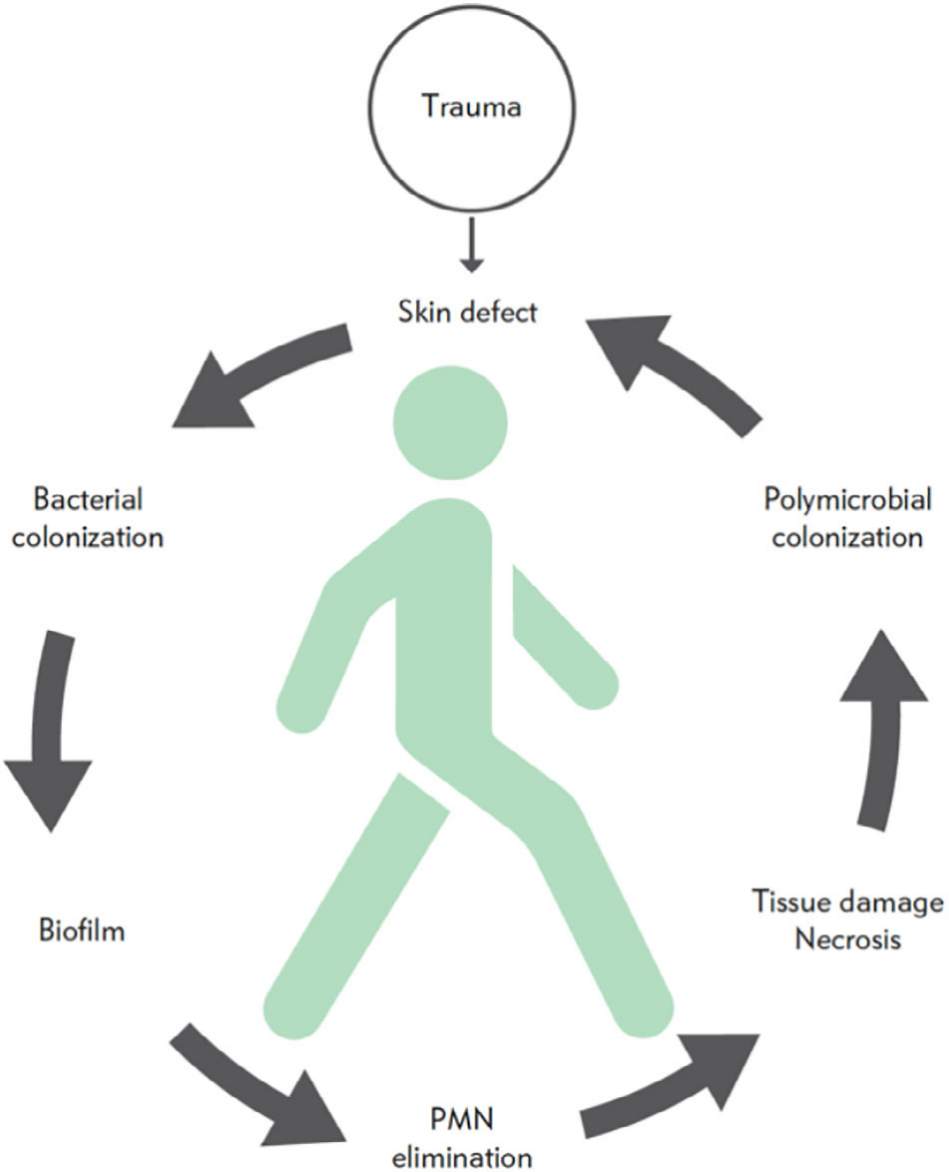
The Wound Treadmill. The treadmill illustrates the relationship between the health of the individual host and the development of a chronic wound. A trauma causes a skin defect that rapidly gets contaminated by bacteria on the skin or in the environment. If they are not cleared from the wound, they will colonize the wound and they will settle in biofilm. Due to the biofilm formation, the bacteria will not be eliminated by the host and may eliminate PMN, causing further tissue damage. The necrotic tissue will facilitate the growth of more bacteria. This vicious circle is illustrated as turning counterclockwise. The person in the center of the treadmill illustrates impact the health status of the host will have on the vicious circle. A healthy host will be able to force the treadmill clockwise, while a host with concomitant diseases will not

Zone 5 is the host. The host may have predisposing factors like diabetes (with or without complications), edema of the lower limbs, vascular impairment, immunologic disturbances, or other contributing localized or general factors. It is rare to encounter chronic wounds in an otherwise healthy host. This is illustrated by the Wound Treadmill (Figure 2). The wound starts with a breach in the skin. This defect is colonized by bacteria, which establish biofilms and thereby cause more tissue damage. The increased tissue damage creates an optimal environment for polymicrobial colonization and further tissue damage. This is challenged by the host, and the health status of the host determines whether the treadmill becomes a vicious circle or moves toward healing. The virulence of the microorganisms also influences the process. This is supported by Gjodsbol's^35^ finding that “…ulcers with *P aeruginosa* were found to be significantly larger than ulcers without the presence of *P aeruginosa*…”.

## 5 UNDERSTANDING THE ZONES AND THEIR RELATIONSHIP

Within the community of a specific biofilm, every bacterium has a unique zone 1 dictating its activity. Therefore, two bacteria within the same biofilm might have markedly different physiological states. Oxygen is a critical electron acceptor for many bacteria. Oxygen is used as an illustrative example to explain the interaction between the zones. The influx of oxygen can, under normal conditions, be regulated by vasodilation when required (seen as erythema in infectious skin conditions). The regulation aims to keep the tissue oxygenated in zone 4. In zone 3, the immune response is mainly driven by PMNs, and their oxidative bursts consume oxygen, decreasing the oxygen available for zone 2. Different aggregates in the same wound can thus have different levels of access to oxygen (in this case due to uneven distribution of PMN in the wound tissue). If the host has ischemia, zone 3 will have less oxygen available, the oxidative burst will be constrained, and zone 2 will have even less oxygen. If the blood supply is restored, this will influence all compartments, and this is seen in clinical cases after by‐pass surgery where infections/inflammation are aggravated. Hyperbaric oxygen therapy can increase the availability of diffused oxygen to zone 4 (and thus all zones). As seen in this example, the zones will influence the adjacent zones reciprocally. Zone 1 will influence zone 2, which is also influenced by zone 3.

Chronic wounds are not normally seen in otherwise healthy persons.^36^, ^37^ Diabetes affects healing on many levels and serves as an example of the complexity of co‐morbidity interference with wound healing. Diabetes causes low‐grade inflammation, local hypoxic conditions, and impaired cellular responses to hypoxia and infection.^38^ As such, a person with diabetes will react differently from a person without diabetes, and their zone 5 will respond differently to challenges.

## 6 UNDERSTANDING BIOFILMS USING THE ZONE MODEL

The zone model may help us understand the “window of opportunity” proposed by Wolcott et al.^39^ It is suggested that surgical debridement of chronic wounds may open a window of opportunity in time during which the bacteria are more susceptible to antibiotics and to the host's immune defense system. They state that it is the physical disruption of the biofilm, and the reattachment to the wound surface, that increase metabolic rate. Through revision, slough, debris, and granulation tissue are removed. To some extent, the bacteria in the superficial layer are also removed. In the context of our zone model, a wound revision induces a change in the environment of zone 4, and this may have a ripple effect on all the zones, forcing/driving the bacteria to alter phenotype or expression. During this adaptation to the new environment, the bacteria (even in biofilm) may be rendered susceptible to antibiotics and accessible to the immune defense system. Treatment of chronic wounds, based on understanding of biofilm, targeted and personalized antimicrobials, and antiseptics, has shown a better outcome compared to historical similar cohorts.^17^

The zone model helps us to understand the different in vitro and animal models and their application for wound biofilm research. In these models, only a limited number of parameters are potentially controllable, such as temperature, oxygen, trace elements, nutrition, selected compounds, treatments, etc. For example, using diabetic animal models, the blood glucose level can be manipulated to a certain extent. It is only zone 4 (and in animal models, zone 5) that can be influenced; as the complexity of the models increase, the less reproduceable the models are.

To illustrate the difference between the flow‐cell biofilm and the in vivo biofilm, consider zone 3 (the “Combat Zone”). In the flow‐cell reactor, zone 3 corresponds to the solution flowing through the reactor. An antibiotic can be added to this solution, but the complexity of the in vivo zone 3 can by no means be reproduced. Furthermore, in the flow‐cell and in most other in vitro models, the bacteria are directly exposed to growth media (including any added antimicrobial agents). In vivo, bacteria are embedded in the wound bed, and thus antimicrobial agents must penetrate either from the blood or topically through zones 3 and 4. Furthermore, in in vitro models, the gradients of oxygen and nutrients are established by a complex interaction between bacteria, host cells, and host extracellular materials.^40^

In terms of animal models, a disadvantage of existing models for diabetic foot ulcers or venous leg ulcers is the lack of contributing factors that are often present in human. Diabetic models do not have micro‐vascular impairment or repetitive pressure on the lesion. Venous leg ulcer models do not have the skin changes that follow many years of chronic edema or impaired lymph drainage. Such factors impinge on zone 4 in animal models, which does not match the real‐life patient situation. A recent article by Cornforth et al^18^ investigated the expression of genes in different infections by *P aeruginosa*. Cystic fibrosis, chronic wounds, diabetic foot ulcers, and burns were compared with in vitro situations and a murine dermal wound model. The expression profile of the bacteria differed between in vitro and in vivo and the patterns of expression in animal models did not match those in humans. This confirms that bacteria respond to the specific microenvironment by activating specific gene sets. Thus, the biochemical at the level of zones 1 to 3 play a significant role.

When we are aiming at introducing a change in the microenvironment, in fundamental research or in clinical research, we can do this in zone 3. The actual change in zone 2 and zone 1 is not known at present. We can investigate the effect on the bacteria with metabolomics, proteomics or RNA sequencing. Yet, these, at least presently, will reflect the average of many bacteria and the change of a single bacteria cannot be detected.

The change we introduce may be temporarily or permanent. Hyperbaric oxygen treatment of infections is an example of introducing a change in zone 5, influencing all zones down to zone 1. The change is temporary, yet some permanent changes may occur. The change in the bacterial phenotype, directly influenced by the change in oxygen levels, is also temporary. The change in phenotype does, however, change the microenvironment and this may have longer effect than the change in oxygen level.

This is highly speculative and needs further research. But without such research the biofilms may render a black box in chronic wound infections.

## 7 FUTURE PERSPECTIVES

In order to understand the behavior and community of bacteria in biofilms, we need to change our view on the current models. We have to acknowledge that every bacterium has a unique microenvironment to which it has a specific response. We have to understand this environment and the responses of different bacteria in order to make progress in the fight against chronic infections.

Equally, it is important to mention the importance of the interaction between the host and microbe and to realize this relates to the micro‐environment too: Every single PMN also reacts to its specific microenvironment as the microorganisms.

The study of the microenvironment, in this context, in zone 1 and 2 calls for development and refinement of research modalities like micro‐sampling, micro‐dialysis and micro‐sensors. Exploring the microenvironment without changing it is crucial.

## 8 CONCLUSION

Despite the fact that bacterial biofilms in chronic wounds have been studied for more than 10 years, we know very little about the microenvironment that surrounds bacteria in chronic infections. Much of our knowledge and conceptual constructs about bacterial biofilms in chronic infections is extrapolated from in vitro biofilm models, yet we know that the bacteria are highly plastic and that the in vitro approximation is incomplete. The vicinity of the bacterial biofilm is poorly investigated and understood in chronic infections. In this article, we have introduced the zone model. We propose that, at present, bacteria in chronic infections are understood only by investigating zone 4, based on examining wound fluid and the knowledge of how bacteria behave in vitro. In order to study the interaction between bacteria and the host we need to look at all environments, as laid out in the zone model.

We hope that the zone model will enable relevant research, improved understanding of bacterial behavior, and development of enhanced models and more effective treatments. Furthermore, we propose that the use of the word “biofilm” should be used only to describe the morphology. We suggest the terminology “bacterial phenotype” be used in order to identify and describe the activity of the bacteria. There are numerous phenotypes of the same species in chronic wound infections, each with different impact on the microenvironment. We can only understand the impact of the presence of biofilm in wounds if we understand the actual metabolism, virulence factor elaboration, antimicrobial susceptibility, and other concrete functions of individual bacteria. This understanding will provide us with the knowledge to successfully overcome the detrimental effect of bacteria in chronic wounds.

In the introduction we, rhetorically, asked the question why the identification of bacterial biofilms in chronic wounds have not led to an improved treatment outcome. The answer may be that we do not know when to and how to eliminate bacterial biofilms. Future research may provide us with a better understanding of the microenvironment and a toolbox for a better outcome.

The zone model needs validation. However, it re‐introduces the single bacterium as the research unit. We need to acknowledge that the wound fluid and tissue specimens, when investigated, represent an average of what is happening in the chronic wound. Hopefully the zone model will help in designing relevant studies of the different zones and their interaction.

## CONFLICT OF INTEREST

The authors declares that they have no conflicts of interest for this article.
